# Estimated breeding values of dairy sires for cow colostrum and transfer of passive immunity traits

**DOI:** 10.3168/jdsc.2024-0575

**Published:** 2024-07-20

**Authors:** A. Soufleri, G. Banos, N. Panousis, V. Tsiamadis, A. Kougioumtzis, G. Arsenos, G.E. Valergakis

**Affiliations:** 1 Laboratory of Animal Husbandry, Faculty of Veterinary Medicine, School of Health Sciences, Aristotle University of Thessaloniki, Greece PC 54124; 2 Scotland's Rural College (SRUC), Easter Bush, Edinburgh, Scotland, UK EH25 9RG; 3 Clinic of Farm Animals, Faculty of Veterinary Medicine, School of Health Sciences, Aristotle University of Thessaloniki, Greece PC 54627

## Abstract

The objective of this study was to derive the estimated breeding values (EBVs) of Holstein sires for colostrum and passive transfer of immunity traits to (1) estimate the genetic association between these new traits and established production, conformation, and function, and (2) explore whether sires can be classified in specific profiles regarding the new traits. For cow colostrum traits, the study included 699 daughters of 67 sires from 6 commercial dairy herds. The number of daughters per sire ranged from 5 to 49. Passive transfer of immunity was measured as the blood serum total protein content in 854 purebred Holstein calves of 61 sires from 8 commercial dairy herds. The number of calves per sire ranged from 5 to 44. Data were statistically analyzed using mixed models. Approximate genetic correlations of the derived sire EBVs for cow colostrum and calf serum total protein with EBVs for several other traits were estimated. Moreover, sires were classified into colostrum and calf serum total protein profile groups. Approximate genetic correlations of cow colostrum and calf serum traits with milk production, conformation, and functional traits were mostly unfavorable. Colostrum TS and protein contents were negatively correlated with milk yield, fat yield, protein yield, productive life, and livability. The only favorable correlation found was between colostrum TS/calf serum total protein and daughter pregnancy rate. However, several bulls had favorable profiles in both colostrum/passive transfer of immunity and production traits. Colostrum/passive immunity traits could be included in future genetic improvement programs after careful structure of relevant indexes.

## Highlights

• The variability in sire EBVs supports the concept of genetic selection.
• Genetic correlations between novel and established traits were unfavorable.
• Bulls with EBV-positive colostrum and production traits were detected.
• A synthetic colostrum quality and passive transfer index could be developed.

## Graphical Abstract

**Figure:**
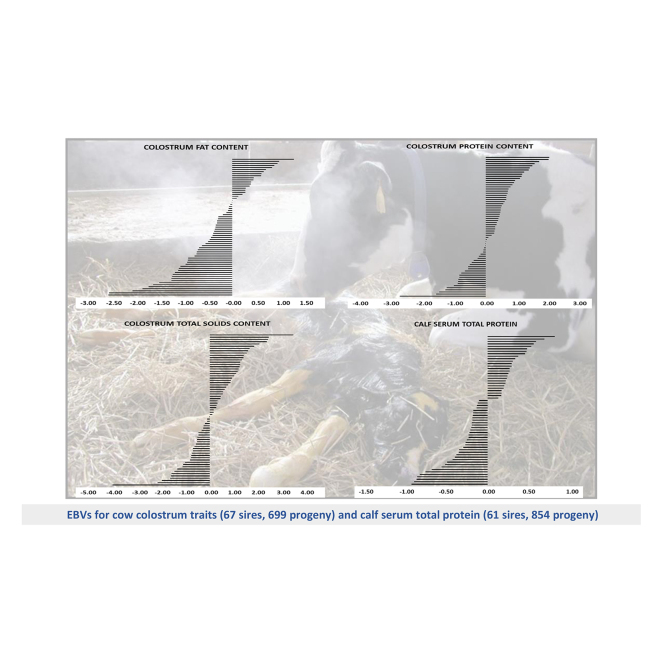
**Summary:** Approximate genetic correlations were estimated, and sire estimated breeding values (EBVs) were calculated for colostrum composition and passive transfer of immunity, which constitute novel traits of calf health and welfare. Sire EBVs were also compared with EBVs for milk production and other traits. Despite the unfavorable genetic correlations detected, several bulls had overall desirable profiles, suggesting the potential inclusion of new traits in future genetic improvement programs.

Dairy cattle selection initially focused on improving milk yield and components; later, conformation and, more recently, traits described as functional, including fertility, health, and longevity, were added to the breeding goals. In a comprehensive review of the genetic selection of economically important traits in dairy cattle, Miglior et al. (2017) proposed several novel traits, including feed efficiency, methane emissions, heat stress adaptation, hoof health, immune response, milk technological properties, and reproduction technology traits. However, there are additional traits of importance associated with animal health and welfare, productivity, and herd sustainability (Weaver et al., 2000; Raboisson et al., 2016) that are not included in the current breeding goals, such as colostrum quality and transfer of passive immunity from cows to neonatal calves. Colostrum is the first secretion of cow mammary gland after parturition, which contains immunoglobulins and important nutritional, growth, and antimicrobial factors (Jaster 2005). Colostrum quality is defined by its immunoglobulin concentration and, therefore, its protein content. Administration of adequate quantities of high-quality colostrum immediately after calving to newborn calves leads to the successful transfer of passive immunity (McGuirk and Collins, 2004) and provides them with important nutrients (Weaver et al., 2000). Fat, the main energy source for newborns, can also be considered a quality factor (Morrill et al., 2012).

Previous genetic studies of colostrum traits have confirmed the presence of heritable variation and, consequently, the possibility of genetically improving these traits via selective breeding. The reported heritability estimates of colostrum fat, protein, lactose, and TS content of Holstein dairy cows ranged from 0.15 to 0.27 (Soufleri et al., 2019; Costa et al., 2022). Moreover, reported statistically significant heritability estimates of calf serum total protein content ranged from 0.05 to 0.21 (Johnston et al., 2020; Soufleri et al., 2023).

Selective breeding is traditionally based on EBVs resulting from the joint analysis of relevant animal phenotypic and pedigree data, after correcting for environmental and other nongenetic factors. The objective of the present study was to derive EBVs for traits related to cow colostrum quality and passive transfer of immunity to calves in a group of Holstein sires-to (1) estimate the genetic association between these new traits and established production, conformation, and functional traits, and (2) explore whether sires can be classified in specific profiles regarding the new traits.

For colostrum traits, the study included 699 milking daughters of 67 sires raised in 6 commercial dairy herds in Greece. For the calf trait, the study included 854 calves of 61 sires, raised in 8 commercial dairy herds in Greece. Number of daughters per sire ranged from 5 to 49 and from 5 to 44, respectively. There were no common sires in the colostrum and calf groups. Cows, calves, and their sires were all purebred Holsteins. Pedigree information spanning 6 ancestral generations was available for all the cows and calves. All studied animals were born through AI with imported semen, mainly from the United States. Herd size ranged from 90 to 300 milking cows. The study was conducted according to the guidelines of the Declaration of Helsinki and approved by the Research Committee of the Aristotle University of Thessaloniki, Greece (Approval protocol number: 50/17.02.2015). Colostrum and serum collection methods have been described in detail by Soufleri et al. (2019) and Soufleri et al. (2023).

Only colostrum samples with normal appearance were collected from clinically healthy cows. Samples were collected by the first author (qualified veterinarian), who also performed a clinical examination on d 1 after calving. Body condition scores were recorded simultaneously using a 1- to 5-point scale (Ferguson et al., 1994) in increments of 0.25. All 4 quarters were completely machine-milked from 10 to 960 min after calving; colostrum yield and the time interval between calving and first milking were recorded. On-farm colostrum quality was determined immediately after sampling using a digital Brix refractometer (PAL-1; Atago, Japan). Colostrum fat, protein, and lactose contents were determined using an infrared milk analyzer (MilkoScan Minor, Foss, Denmark) at the Laboratory of Milk Hygiene and Technology of the Faculty of Veterinary Medicine, Aristotle University of Thessaloniki.

All calves were fed their dam's colostrum and were clinically examined by the first author 24 to 48 h after birth. If found healthy, a blood sample was collected at the same time by jugular venipuncture. Calf BW was estimated using a heart girth tape, and the quality and quantity of colostrum fed and the time interval between birth and colostrum administration were recorded. Blood samples were centrifuged (4,000 × *g* for 15 min at 20°C). In the present study, calf serum total protein content was measured using the same digital Brix refractometer and constituted the transfer of passive immunity trait.

Cow colostrum and calf serum traits were analyzed using the following mixed model:

Y=Xb+Zu+e,

where *Y* is the animal record for each trait (colostrum TS, fat, protein, lactose content, and calf serum total protein content, dependent variable); **b** is the vector of fixed effects, described below; **u** is the vector of random animal additive genetic effects, ∼*N* $\left(0,A\sigma_{a}^{2}\right),$ where A is the pedigree relationship matrix and $\sigma_{a}^{2}$ is the additive genetic variance estimate; **e** is the vector of random residual effects, ∼*N* $\left(0,I\sigma_{e}^{2}\right),$ where **I** is the identity matrix and $\sigma_{e}^{2}$ is the residual variance; **X** and **Z** are the incidence matrices linking the observations to fixed and random additive genetic effects, respectively.

For the colostrum traits, fixed effects included dry period length, milk yield of previous 305-d lactation, time interval between calving and colostrum collection, BCS, and age at calving as covariates and farm, parity number, season of calving, and colostrum yield as categorical variables. Parity number was grouped as 1, 2, 3, and ≥4. Season of calving was categorized as follows: winter (December, January, February), spring (March, April, May), summer (June, July, August), and autumn (September, October, and November). Colostrum yield was classified as follows: ≤4, 4.1–8.4, and ≥8.5 kg.

For calf serum protein content, fixed effects included BW, quality and quantity of colostrum fed, time interval between birth and colostrum administration, time interval between birth and blood sampling (as covariates), and farm, calendar year-season of birth, calf sex, and calving ease (as categorical variables). Season of birth was categorized as previously described for season of calving. Calving ease was categorized as 1, 2, 3, 4, and 5 (1: no assistance and 5: abnormal delivery).

The fixed effects were tested in preliminary analyses to confirm whether there was a statistically significant effect (*P* < 0.05) on the traits. Milk yield of the previous 305-d lactation and BCS did not have such an impact and were not included in the final colostrum model. For this reason, calf sex, BW, and calving ease were not included in the final calf serum model. Software package ASREML (Gilmour et al., 2009) was used for all the analyses.

Sire EBVs were derived directly from the solutions of the random animal genetic effect in the above models. Reliability was calculated as described by Onyiro et al. (2008).

Descriptive statistics of the derived sire EBVs for colostrum and calf serum traits are presented in Table 1. Estimated breeding values and their reliabilities for milk production, conformation, and functional traits of the sires included in the present study were obtained online from the DairyBulls.com website (US genetic base, April 2023 evaluation). Traits included milk, fat, and protein yield (**MY**, **FY**, and **PY**, respectively), milk fat and protein content (**FC** and **PC**, respectively), productive life (**PL**), SCS, daughter pregnancy rate (**DPR**), livability (**LIV**), udder composite (**UC**), and feet and legs composite. Correlations between the latter EBVs and cow colostrum and calf serum trait EBVs derived in the present study were estimated. Notably, EBVs for the novel traits were based on sire progeny in Greece and were not used in derivation of the other trait EBVs, which were based on US data. Therefore, the 2 datasets were independent and suitable for calculating-approximate genetic correlations between traits. These correlations were estimated using the following equation (Calo et al., 1973):

$$rg_{1,2}=\frac{\sqrt{\left(\Sigma \;Rel_{1}\right)\times \left(\Sigma \;Rel_{2}\right)}}{\Sigma \;\left(Rel_{1}\times Rel_{2}\right)}\times r_{1,2},$$

where r*g*_1,2_ = approximate genetic correlation between traits 1 and 2; *Rel*_1_ and *Rel*_2_ = reliabilities of EBVs for traits 1 and 2; and r_1,2_ = correlation between EBVs for traits 1 and 2. Approximate standard errors of genetic correlations were estimated as follows (Onyiro et al., 2008):

$$SE=\sqrt{\left(1-r^{2}g_{1,2}\right)/n-2\;},$$

where *n* = number of sires with records. Subsequently, sires were classified into the following groups: (A) overall positive colostrum profile: bulls with positive EBVs for colostrum fat and TS content; (B) positive immunological colostrum profile: bulls with positive EBVs for colostrum TS content and negative EBVs for colostrum fat content; (C) positive fat colostrum profile: bulls with positive EBVs for colostrum fat content and negative EBVs for colostrum TS content; and (D) overall negative colostrum profile: bulls with negative EBVs for colostrum fat and TS content. Regarding calf serum total protein trait, bulls were categorized into 2 groups: (A) positive calf serum total protein profile and (B) negative calf serum total protein profile.

**Table 1:** Descriptive statistics of sire EBVs and corresponding progeny phenotypes for cow colostrum traits (67 sires, 699 progeny) and calf serum total protein (61 sires, 854 progeny)

Trait	EBV				Progeny phenotype: average ± SE
	Average ± SE	10th percentile^1^ upper limit, average ± SE	90th percentile^2^ lower limit, average ± SE	Average EBV reliability	
Colostrum fat content (%)	−0.40 ± 0.10	−1.56	+0.67	0.29	6.40 ± 0.10
		−2.09 ± 0.17	0.94 ± 0.08		
Colostrum protein content (%)	+0.19 ± 0.11	−0.92	+1.40	0.34	17.80 ± 0.12
		−1.57 ± 0.25	+1.71 ± 0.11		
Colostrum lactose content (%)	−0.03 ± 0.02	−0.21	+0.12	0.24	2.20 ± 0.02
		−0.26 ± 0.02	+0.26 ± 0.04		
Colostrum TS (% Brix value)	+0.17 ± 0.16	−1.50	+1.70	0.42	25.80 ± 0.14
		−2.18 ± 0.40	+2.44 ± 0.23		
Calf serum total protein (% Brix value)	−0.07 ± 0.05	−0.75	+0.48	0.33	8.50 ± 0.04
		−0.86 ± 0.02	+0.62 ± 0.05		

1 10th percentile represents 10% of lower-ranking bulls. Upper limit denotes that all of these bulls had EBVs less than or equal to the corresponding values. Average ± SE represent EBVs of the percentile.

2 90th percentile represents 10% of higher-ranking bulls. Lower limit denotes that all of these bulls had EBVs greater than or equal to the corresponding values. Average ± SE represent EBVs of the percentile.

Approximate genetic correlations of cow colostrum and calf serum traits in Greece with milk production, conformation, and functional traits in the United States are presented in Table 2. Colostrum TS content was found to be unfavorably correlated with MY, FY, PY, and LIV; these correlations were statistically significant (*P* ≤ 0.05). Correlation of colostrum protein content with MY, PY, and longevity traits (PL and LIV) followed a similar pattern. Interestingly, unfavorable and statistically significant correlations were found between colostrum fat content and FY and FC, as well as with UC (*P* ≤ 0.05). Colostrum lactose content was positively and significantly correlated with FY, PY, PC, PL, and LIV; however, these correlations were unfavorable because it was negatively correlated with TS content (Soufleri et al., 2019), the main practical quality criterion of colostrum. Overall, colostrum quality traits were unfavorably correlated with production and longevity traits. The only favorable and statistically significant correlation was found between colostrum TS and DPR (*P* ≤ 0.05). The latter was the only trait correlated with calf serum total protein content, again in a favorable mode.

**Table 2:** Approximate genetic correlations ± SE of cow colostrum and calf serum total protein with milk production, conformation, and functional traits

Trait	Colostrum TS	Colostrum fat content	Colostrum protein content	Colostrum lactose content	Calf serum total protein
Milk yield	−0.56 ± 0.10*	−0.18 ± 0.12	−0.49 ± 0.11*	+0.20 ± 0.12	−0.16 ± 0.13
Milk fat yield	−0.30 ± 0.12*	−0.41 ± 0.11*	−0.15 ± 0.12	+0.31 ± 0.12*	+0.10 ± 0.13
Milk fat content	+0.09 ± 0.12	−0.26 ± 0.12*	+0.17 ± 0.12	+0.17 ± 0.12	+0.02 ± 0.13
Milk protein yield	−0.50 ± 0.11*	−0.12 ± 0.12	−0.49 ± 0.11*	+0.37 ± 0.12*	+0.16 ± 0.13
Milk protein content	−0.15 ± 0.12	+0.05 ± 0.12	−0.21 ± 0.12	+0.29 ± 0.12*	+0.01 ± 0.13
Productive life	−0.21 ± 0.12	−0.15 ± 0.12	−0.24 ± 0.12*	+0.33 ± 0.12*	+0.17 ± 0.13
SCS	−0.13 ± 0.12	−0.14 ± 0.12	−0.10 ± 0.12	−0.05 ± 0.12	−0.03 ± 0.13
Daughter pregnancy rate	+0.27 ± 0.12*	+0.21 ± 0.12	+0.22 ± 0.12	−0.04 ± 0.12	+0.33 ± 0.12*
Livability	−0.41 ± 0.11*	−0.12 ± 0.12	−0.45 ± 0.11*	+0.39 ± 0.11*	−0.09 ± 0.13
Udder composite	−0.07 ± 0.12	−0.39 ± 0.11*	−0.06 ± 0.12	+0.17 ± 0.12	+0.07 ± 0.13
Feet and legs composite	−0.06 ± 0.12	−0.16 ± 0.12	−0.11 ± 0.12	+0.11 ± 0.12	−0.11 ± 0.13

* *P* ≤ 0.05.

The profile of bull EBVs for colostrum traits was generally consistent with the correlations presented above. However, there are a few notable exceptions. Group A (n = 14) that had an overall positive colostrum profile, and therefore expected to be associated with reduced production, included 4 bulls that had positive EBVs for milk (+537 ± 181 kg), fat (+9.0 ± 2.8 kg), and protein (+14 ± 2.7 kg) yield and 2 bulls with positive EBVs for milk (+362 ± 26 kg) and protein (+1.6 ± 0.9 kg) yield. Bulls in group B (n = 22) that had positive EBVs for TS and negative for colostrum fat were expected to have negative EBVs for milk and protein yield. However, 10 of these bulls had positive EBVs for milk (+591 ± 147 kg), especially for protein (+23.7 ± 7.6 kg) yield. Bulls in group C (n = 7) that had negative EBVs for TS and positive for colostrum fat were expected to have positive EBVs for production traits. However, 2 bulls had negative EBVs for milk (−451 ± 35 kg), fat (−5 ± 4.6 kg), and protein (−11.8 ± 2.4 kg) yield. Group D (n = 24) that had a negative colostrum profile, and therefore was expected to have positive production, included 10 bulls that had negative EBVs for milk fat (−11.1 ± 3.4 kg) yield and neutral EBVs for protein (+0.1 ± 1.7 kg) yield.

Of the 26 bulls classified as having a positive calf serum total protein profile, 18 had positive EBVs for MY and PY and 8 bulls had negative ones. Of the 35 bulls with negative calf serum total protein profiles, 30 had positive EBVs for MY and PY, and 5 bulls had negative ones. Overall FY was positive, but bulls with negative EBVs were present.

Sire EBVs for colostrum and calf traits and their variabilities are reported here for the first time.

Despite the relatively small number of bulls, compared with the global inventory of Holstein sires used in AI, the present study revealed substantial variation in their genetic potential in terms of their daughters' colostrum quality traits, which was also evident from their progeny phenotypes.

Genetic correlations between traits reported here suggest that continuous selection for higher yield (MY, FY, and PY) and longevity (PL and LIV) would lead to a deterioration of colostrum quality, manifested in lower TS and protein content. Since milk yield improvement is the main criterion that farmers use in bull selection (Egger-Danner et al., 2015; Cole et al., 2023), and both yield and longevity are considered as major short- and long-term profitability factors (Miglior et al., 2017), over the years, this has probably already had an undesirable side effect on colostrum quality. Moreover, selection for milk fat yield, which over the past 10 years has led to an increase of 0.17 percentage units and 53.1 kg (Amaral-Phillips, 2020) and improved udder conformation might have resulted in compromised colostrum fat content. The latter is particularly critical for neonates to combat cold stress.

In contrast, based on our findings, a favorable effect on colostrum TS content would result when selecting for improved DPR; the former had a significant correlation with calf serum total protein, as well, and was improved by 7% from 2010 to 2015 (García-Ruiz et al., 2016) despite having a modest weight in the selection indexes (7%–11% Net Merit$) (VanRaden et al., 2021). Therefore, it is probable that any undesirable side effect on colostrum quality previously described may have been partly alleviated by selection for improved fertility. There is no biological explanation for the favorable association between passive transfer of immunity and DPR. Adequate transfer of passive immunity leads to reduced morbidity, and therefore, healthier calves and heifers with sufficient growth rates, which are presumably more fertile. Wathes et al. (2008) reported that heifers with low growth rates required more services to conceive, had a higher age at first calving, and calved later compared with heifers with optimal growth. However, DeNise et al. (1989) failed to show an effect of calf serum immunoglobulin content on age at first calving.

Colostrum and calf serum phenotypes in the present study were collected in Greece, whereas EBVs of the studied sires for the other traits were obtained from the national genetic evaluation in the United States. Therefore, the genetic correlations between traits discussed above may be affected by possible genotype-by-country interactions. The latter is a consequence of sire selection transcending national borders and features in the international genetic evaluations of dairy sires produced by the International Bull Evaluation Service (Interbull, 2023). In these evaluations, genotype-by-country interactions were addressed by the genetic correlation of a trait expressed on the scale of different countries. Data from Greece are not included in the Interbull evaluations; therefore, correlations between Greece and the United States are not available. Therefore, in the present study, genetic correlation was approximated using the method described by Calo et al. (1973), further exemplified in previous studies of data from different countries (Rogers et al., 1998, 1999). Subsequently, we used the correlation between Italy and the United States for milk production, conformation, and functional traits as a proxy for the correlation between Greece and the United States for these traits. The latter is supported by geographic proximity, and similarities in climate and production systems (climatic conditions and housing, feeding system and general management practices) between Greece and Italy. Genetic correlations between Italy and the United States were quite high, ranging from 76% to 96%. Therefore, we believe that the actual genetic correlations of colostrum and passive immunity traits with milk production, conformation, and functional traits are likely to remain in the ballpark of estimates shown in Table 2.

In this study, bulls were classified according to their colostrum profiles and their respective EBVs for milk production traits. Despite the overall antagonistic genetic correlations between the 2 sets of traits, approximately 24% of the sires still combined all desirable traits and approximately 18% had all undesirable traits. Regarding calf serum total protein content, the issue of sire selection appears relatively simpler, as approximately 30% of the bulls combined all desirable traits.

Even if the genetic correlation between 2 traits is antagonistic, selection for both traits is still possible (Oldenbroek and Van der Waaij, 2015), if based on a carefully constructed index, the trade-off is a slower genetic response on each trait separately due to lower selection intensity. Selection based on multiple-trait indices has already been applied successfully for genetically antagonistic traits, such as production versus health and production versus fertility, which are included with different relative emphases in various national selection indices around the world (Miglior et al., 2005, 2012). Genetic progress is expected to continue or at least not deteriorate when selecting for antagonistic traits (VanRaden et al., 2021).

We acknowledge the relatively small data size available for this study as a limitation. Our results must be confirmed by larger-scale studies before inclusion of new traits in genetic improvement schemes. However, despite the evident genetic antagonism of colostrum quality with some of the cow traits in the current breeding goal, we identified individual bulls with desirable EBVs for all traits. Presence of such selection candidates is expected for quantitative traits with complex inheritance and imperfect genetic correlation and underpins selective breeding for multiple traits using carefully built selection indices. Future genomic and transcriptomic studies would elucidate the action and interaction between the genes involved and shed light on the molecular profile of the studied traits. Upon confirmation of our results and after estimating the economic weight of colostrum and passive immunity transfer traits, a synthetic “passive immunity” index could be developed and eventually included in an overall performance index in future breeding programs.
